# Roles and impacts of pharmacists in attention-deficit hyperactivity disorder services: a scoping review

**DOI:** 10.1007/s11096-026-02184-2

**Published:** 2026-07-15

**Authors:** Muhammad Umair Khan, Atta Abbas Naqvi, Ian Chi-Kei Wong, Ian Maidment, Graham Brown, Samuele Cortese

**Affiliations:** 1 Aston Pharmacy School, School of Medicine, Pharmacy and Biosciences, Birmingham, UK; 2 School of Pharmacy, University of Reading, Whiteknights Campus, Reading, UK; 3 Centre for Safe Medication Practice and Research, Department of Pharmacology and Pharmacy, Li Ka Shing Faculty of Medicine, The University of Hong Kong, Pok Fu Lam, Hong Kong Special Administrative Region China; 4 School of Pharmacy, Medical Sciences Division, Macau University of Science and Technology, Taipa, Macau Special Administrative Region China; 5 Advanced Data Analytics for Medical Science Limited, Hong Kong Special Administrative Region, China; 6 Hampshire and Isle of Wight Healthcare NHS Foundation Trust, Southampton, UK; 7 Developmental EPI (Evidence synthesis, Prediction, Implementation) Lab, Centre for Innovation in Mental Health, School of Psychology, Faculty of Environmental and Life Sciences, University of Southampton, Southampton, UK

**Keywords:** ADHD, Medicine optimisation, Pharmacist, Prescribing, Service delivery

## Abstract

**Introduction:**

Demand for attention-deficit hyperactivity disorder (ADHD) services has increased substantially across health systems, placing pressure on specialist services and contributing to prolonged waiting times and inconsistent medication monitoring. Pharmacists have established expertise in medicines optimisation and, in some settings, are authorised to prescribe. Yet their role within ADHD care pathways remains poorly characterised.Kindly check and confirm the inserted city name in affiliations 3, 5, 6 and country name in affiliation 6 are correct and amend if necessary.Changes have been made, where necessary.

**Aim:**

This scoping review aims to map the existing empirical evidence on pharmacist involvement in ADHD services, synthesise reported roles and impacts, and identify key evidence gaps to inform future research and service development.

**Method:**

A scoping review was conducted in accordance with the Arksey and O’Malley framework and reported using PRISMA-ScR guidance. Six databases (PubMed, PsycINFO, Embase, CINAHL, Scopus, and Web of Science) were searched from inception to December 2025. Empirical studies evaluating implemented pharmacist involvement in ADHD services were included, irrespective of study design. Data were charted and synthesised narratively across domains relating to clinical care, service delivery, economic outcomes, and barriers to implementation.

**Results:**

Thirteen studies were included, published between 2008 and 2025, primarily from the United States and the United Kingdom. Most were service evaluations or pre-post studies, with one randomised trial. Pharmacists were involved in a broad range of activities, including medication initiation, titration, monitoring, follow-up, patient education, and service governance, often as independent prescribers or within collaborative multidisciplinary models. Reported impacts included improved adherence to recommended monitoring standards, increased service capacity, reduced waiting times, and more efficient workforce utilisation, with some evidence of potential cost savings. However, outcome measures varied widely, and patient-reported outcomes, comparative effectiveness, and implementation processes were inconsistently examined.

**Conclusion:**

This review identifies a small but expanding evidence base indicating potential roles for pharmacists in ADHD services, particularly in medicines management and service delivery. However, the absence of clearly defined service models and limited comparative and implementation-focused evidence constrain wider adoption across health systems. Further research is needed to inform scalable and sustainable approaches to pharmacist integration in ADHD care.

**Supplementary Information:**

The online version contains supplementary material available at https://doi.org/10.1007/s11096-026-02184-2.

## Impact statements

- This review provides the first comprehensive synthesis of empirical evidence on pharmacist involvement in ADHD services. It demonstrates that pharmacists can contribute effectively to medication initiation, titration, monitoring, and service delivery, with potential benefits for access to care and adherence to recommended monitoring standards.
- Findings suggest that integrating pharmacists into ADHD care pathways may improve service capacity and workforce efficiency, particularly in health systems experiencing workforce shortages and long waiting times. Pharmacist-led or pharmacist-supported models could support more timely access to medication management while allowing specialist clinicians to focus on diagnostic assessment and complex cases.
- Despite emerging evidence supporting pharmacist roles in ADHD care, the literature remains limited and heterogeneous, with few comparative studies and minimal evaluation of patient-reported outcomes or implementation processes. Further robust research is needed to define scalable service models and inform sustainable integration of pharmacists within ADHD services.

## Introduction

Attention-deficit hyperactivity disorder (ADHD) is a common neurodevelopmental condition affecting approximately 8% of children globally [1] and 2.6% of adults [2]. In the United Kingdom (UK), prevalence estimates suggest ADHD affects around 5% of children and 3–4% of adults [3]. ADHD is characterised by inattention, hyperactivity, and impulsivity and is associated with impaired educational attainment, increased psychiatric comorbidity, and long-term socioeconomic disadvantage [4]. Pharmacological treatment, alongside psychosocial interventions, remains a cornerstone of ADHD management for many individuals, with stimulant and non-stimulant medications requiring ongoing clinical review and monitoring [3].

Despite the availability of effective treatments, access to ADHD services remains a significant challenge internationally. Rising demand, workforce shortages, and increasing complexity of care have placed sustained pressure on specialist services [5–7]. In the UK, waiting times for assessment and treatment initiation are frequently prolonged. The NHS England ADHD Taskforce reported waits of up to four years for children and eight years for adults [8], contributing to delayed treatment, inconsistent medication monitoring, and variable patient experience [9]. The requirement for regular physical monitoring further increases service burden and exacerbates inequalities in access [10].

Pharmacists are a highly skilled but underutilised workforce in mental health and neurodevelopmental care [11]. Particularly, specialist and clinically trained pharmacists possess expertise in medicines optimisation, safety monitoring, and patient education, positioning them to support ADHD services. Evidence from broader mental health settings suggests pharmacist involvement can improve medication management and service efficiency [11]. In ADHD care, pharmacists may contribute to medication initiation and titration, routine monitoring, adherence support, and coordination across care settings. However, the scope, implementation, and impact of these roles remain poorly characterised.

Emerging empirical studies, predominantly from the United States (US) and the UK, have evaluated pharmacist-led or pharmacist-supported ADHD services across a range of settings [12–14]. However, this literature varies widely by study design, populations, pharmacists’ role, service model, and the evidence base remains fragmented. It is also unclear to what extent existing studies have examined patient-reported outcomes, clinician perspectives, or implementation challenges relevant to the sustainability and scalability of pharmacist involvement in ADHD services. Existing narrative reviews and book chapters describe potential pharmacist contributions but do not synthesise empirical evaluations of service delivery or outcomes [15–17]. To date, no review has comprehensively mapped the empirical evidence on pharmacist involvement in ADHD services.

### Aim

This scoping review aims to map the existing empirical evidence on pharmacist involvement in ADHD services, synthesise reported roles and impacts, and identify key evidence gaps to inform future research and service development.

## Method

This scoping review was conducted in accordance with the framework proposed by Arksey and O’Malley [18] and informed by subsequent methodological guidance [19] and reported in line with the Preferred Reporting Items for Systematic Reviews and Meta-Analysis extension for Scoping Reviews (PRISMA-ScR) [20]. Although a formal protocol for this scoping review was not registered or published, a structured approach informed by the methodological guidance outlined above was followed in conducting the five stages of the review: (1) identifying the research question, (2) identifying relevant studies, (3) study selection, (4) charting the data, and (5) collating, summarising, and reporting the results.

### Stage 1: identifying the research question

The review was guided by the following research question:

What is the current evidence on the roles and impacts of pharmacists in ADHD services, and what gaps exist to inform future service development?

This broad question was selected to enable mapping of the range, nature, and extent of empirical evidence relating to pharmacist involvement across different ADHD care settings, populations, and service models.

### Stage 2: identification of relevant studies

A comprehensive search strategy was developed and applied across six electronic databases: PubMed, PsycINFO, Embase, CINAHL, Scopus, and Web of Science. Searches were limited to studies published from inception to December 2025 to capture the evolution of pharmacist involvement beyond traditional dispensing roles. Only English-language publications were included.

The search strategy combined terms related to ADHD (e.g. attention deficit hyperactivity disorder, ADHD) with terms describing pharmacists and pharmacy professionals (e.g. pharmacist, clinical pharmacist, specialist pharmacist, independent prescriber) and terms relating to services, roles, and care delivery (e.g. prescribing, titration, monitoring, intervention). Database-specific subject headings (e.g. MeSH terms) and Boolean operators were used to optimise sensitivity and relevance. A sample search strategy is provided in Appendix 1.

In addition to database searching, reference lists of included studies and relevant reviews were hand-searched to identify further eligible articles. Conference proceedings were also screened to capture emerging evidence not yet published in full.

### Stage 3: study selection

Study selection was conducted in two stages. First, titles and abstracts of all identified records were independently screened by two reviewers (MUK & AN) to assess potential eligibility. Discrepancies were resolved through discussion and consensus.

Second, full-text screening was undertaken by one reviewer (MUK) and cross-checked by a second reviewer (AN). Studies were included if they reported on the involvement of a pharmacist in an ADHD service and evaluated the impact of pharmacist activities on patient care, clinical processes, or service delivery. For the purpose of this review, evaluation of impact was interpreted broadly and included both quantitative outcomes (e.g. monitoring rates, waiting times, or prescribing outcomes) and descriptive or service-level evaluations reporting changes in practice, service delivery, or implementation. Studies were excluded if ADHD was not the primary focus, if pharmacist involvement was limited to theoretical or conceptual descriptions without service implementation, or if no evaluation of impact was reported. Conference abstracts were included where sufficient methodological detail and outcome data were available to inform the review. The study selection process was documented using a PRISMA-ScR flow diagram.

### Stage 4: charting the data

Data were charted using a data extraction form and piloted by the review team. Extraction was undertaken primarily by one reviewer (MUK), with all extracted data checked by a second reviewer for accuracy and completeness (AN). Any discrepancies were resolved through discussion and consensus.

Extracted data included study characteristics (author, year, country), study design and duration, care setting, target population, sample size, and pharmacist role (e.g. independent prescriber, specialist pharmacist, collaborative model). Information on service models, additional training, and the number of pharmacists involved was also collected where reported.

Outcome data were extracted verbatim and included clinical and medication management outcomes, service delivery and access outcomes, economic or resource-related outcomes, and system-level or professional contributions. Where reported, information on implementation barriers and facilitators was also charted. Consistent with scoping review methodology, no formal assessment of study quality or risk of bias was undertaken, as the aim was to map the extent, nature, and characteristics of the evidence rather than to evaluate effectiveness [18].

All data were managed using Microsoft Excel.

### Stage 5: collating, summarising, and reporting the results

Extracted data were collated and synthesised using a narrative descriptive approach. Study characteristics were summarised in tabular form, while findings were organised into several domains reflecting the objectives of the review. These domains included clinical and medication management outcomes, service delivery, efficiency, and access to care, economic and resource outcomes, professional contribution and system-level functions, and barriers to implementation and sustainability of pharmacist-led ADHD services.

Given the diversity of study designs, settings, and outcome measures, quantitative data were summarised descriptively, and qualitative findings were synthesised narratively. Results are presented in accordance with PRISMA-ScR guidance (Appendix 2).

## Results

**Fig. 1:**
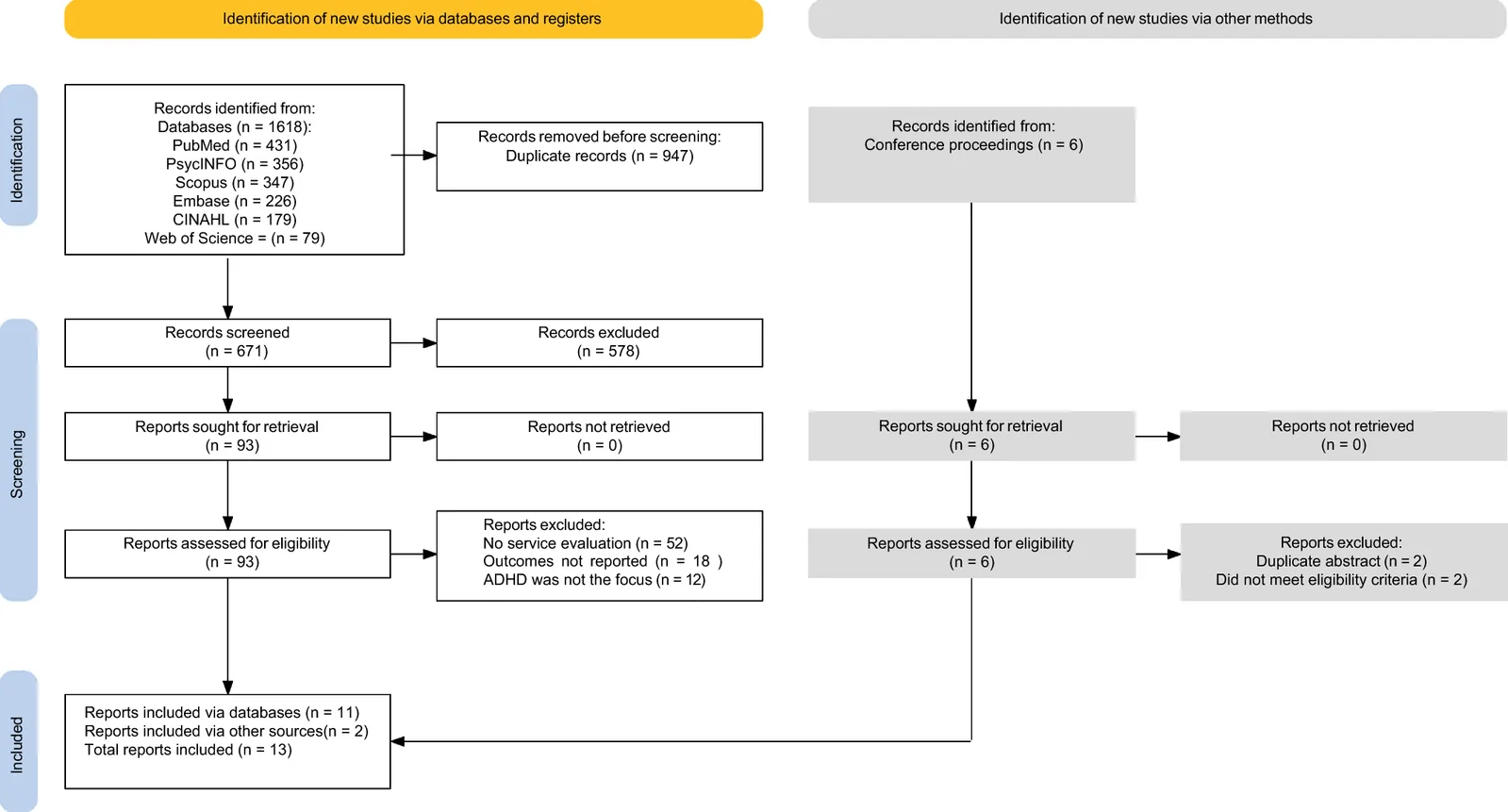
PRISMA flow diagram

The database searches identified 1618 records, with an additional six records identified through other sources (conference proceedings). After the removal of 947 duplicates, 671 records were screened by title and abstract. Of these, 578 records were excluded, and 93 full-text articles were assessed for eligibility. Eighty-two full-text articles were excluded for not meeting the inclusion criteria, resulting in 11 studies included from database searches. Of the six records identified from other sources, four were excluded due to duplication or ineligibility, and two were included. In total, 13 studies were included in the final analysis (Fig. 1).

### Overview of included studies

All included studies were published between 2008 and 2025, with most published after 2015 (n = 11, 84.6%). Studies were conducted in the US (n = 7, 53.8%) [12, 21–26], the UK (n = 5, 38.5%) [13, 14, 27–29] and Thailand (n = 1, 7.7%) [30]. Eight studies employed a prospective design (61.5%) [13, 14, 21, 22, 24, 25, 28, 29], and five were retrospective (38.4%) [12, 20, 23, 26, 27]. Post-implementation evaluations were most common (n = 5, 38.4%) [13, 14, 24, 26, 29], followed by pre-post studies (n = 4, 30.8%) [12, 25, 27, 30], service innovation (n = 2, 15.4%) [21, 23], cross-sectional study (n = 1, 7.7%) [28], and a cluster randomised controlled trial (n = 1, 7.7%) [21]. Study duration varied substantially, ranging from 2 months to 11 years.

Pharmacist numbers ranged from one to seventy-two, and patient sample sizes from 50 to 914. Pharmacist roles included clinical pharmacists (n = 5; 38.5%) [12, 21, 23, 25, 30], independent prescribers (n = 3; 23.1%) [13, 14, 29], specialist mental health pharmacists (n = 3; 23.1%) [22, 24, 27], community pharmacists (n = 1; 7.7%) [28], and mental and behavioural health pharmacists (n = 1; 7.7%) [26].

Target populations included children (n = 7, 53.8%) [13, 14, 21, 26–28, 30], adults (n = 5, 38.5%) [12, 22–24, 29], and school personnel (e.g., school nurse) (n = 1, 7.7%) [25]. Settings included CAMHS (n = 3, 23.1%) [13, 14, 27], adult ADHD services (n = 1, 7.7%) [29], medical centres (n = 4, 30.8%) [21–24], school or college settings (n = 2, 15.4%) [12, 25], tertiary paediatric health system (n = 2, 15.4%) [26, 30], and community pharmacy (n = 1, 7.7%) [28]. Study characteristics are summarised in Table 1.

**Table 1:** Study characteristics

Study (Country)	Study design	Evaluation approach	Sample size	Study duration	Settings	Pharmacist role	Model of care	Additional training	ADHD population	Findings
Alfredson, 2025 (US) [26]	Retrospective	Post-implementation evaluation	Pharmacist = 1 Consultations = 69	12 months	Tertiary paediatric health system	Mental and behavioural health pharmacist	Collaboration with primary care providers	No	Children (< 18 years)	Pharmacists consulted 46.3% of ADHD cases; 97.6% of recommendations accepted. Activities included clinical review, medication access, education, and adherence support
Allen, 2025 (UK) [29]	Prospective	Post-implementation evaluation	Pharmacists = 2	6 months	Adult ADHD Services	Independent prescriber	Pharmacist working independently within multidisciplinary team	Yes (Supervision)	Adults	Pharmacist-led services included consultation, prescribing, documentation, and follow-up planning. Reduced clinician workload through repeat prescribing for stable patients
Casey, 2020 (US) [23]	Retrospective	Service innovation	Pharmacists = 6 Patients = 914	5 years	Medical centre	Clinical pharmacist	Collaboration with psychiatrist and primary care physician	No	Adults	610/914 patients were successfully stabilised by pharmacist-led care, with 2,634 encounters and regular follow-up. Cost savings of $761,280 were reported. Barriers included state-specific prescribing regulations
Huang, 2020 (US) [22]	Prospective	Service innovation	N/A	N/A	Medical centre	Specialist mental health pharmacist	Collaboration with physician and psychiatrist	No	Adults	Development of a standardised protocol for assessment, referral, and follow-up, including medication monitoring, titration, and use of screening measures. Reduced caseload for other practitioners
Lee, 2020 (US) [24]	Prospective	Post-implementation evaluation	Pharmacists = 1 Patients = 58	3 years	Medical centre	Specialist mental health pharmacist	Collaboration with psychiatrist	No	Adults	774 patient encounters recorded. Pharmacist roles included psychiatric evaluation, medication management, counselling, and referral. Barriers included limited time, prescribing authority, and reimbursement constraints
Lieu, 2025 (US) [21]	Prospective	Cluster RCT	Pharmacist care = 512 Paediatrician care = 1930	2 months	Medical centre	Clinical pharmacist	Collaboration between pharmacist and paediatrician	No	Children (< 18 years)	Higher adherence to height and weight monitoring with pharmacist care (88.6% vs 66.1%). Prescription ordering time slightly longer, but medication filling time and parent-rated quality were similar
Lyon, 2018 (UK) [27]	Retrospective	Pre-post study	Pharmacists = 1	11 years	CAMHS	Specialist mental health pharmacist	Collaboration with medical staff	No	Children (< 18 years)	Annual drug cost reduction of approximately £133,000, with net savings of £97,000. Pharmacists supported medicines optimisation, guideline development, clinician training, and service governance
O’Brien, 2021 (UK) [14]	Prospective	Post-implementation evaluation	Pharmacists = 1 Patients = 78	6 months	CAMHS	Independent prescriber	Independent prescribing	Yes (8 weeks)	Children (< 18 years)	28 patients (36%) initiated on ADHD medication, with most responding to first-line treatment. Waiting time reduced from 7 to 3 months. Contributions included clinical governance support and management of high-risk medication
Pohl, 2021 (US) [12]	Retrospective	Pre-post study	Pharmacist = 1 Patients = 481	3 years	College health centre	Clinical pharmacist	Collaboration followed by independence	No	Adults	Pharmacist FTE increased from 0 to 0.2, with a 1003% rise in appointments (26 to 287). Improved adherence to blood pressure and heart rate monitoring compared with psychiatrist-led care. Limitation included lack of structured service templates
Reutzel, 2008 (US) [25]	Prospective	Pre-post study	Pharmacists = 2 School staff = 321	N/A	School	Clinical pharmacist	Collaboration with mental health therapists	No	School personnel	Educational sessions improved knowledge and confidence among school personnel regarding ADHD. Supported wider education for staff, families, and students through collaboration with health professionals
Shah, 2021 (UK) [13]	Prospective	Post-implementation evaluation	Pharmacist = 2 Patients = 50	3 months	CAMHS	Independent prescriber	Independent prescribing	Yes (6 months)	Children (< 18 years)	322 pharmacist contributions including prescribing support, wellbeing assessment, physical monitoring, medicines advice, and caseload management
Tobaiqy, 2010 (UK) [28]	Prospective	Cross-sectional survey	Pharmacists = 72	3 months	Community pharmacy	Community pharmacist	Pharmacist working independently	No	Children (< 16 years)	26 adverse drug reactions reported, most commonly appetite loss, psychiatric symptoms, gastrointestinal disturbance, sleep disturbance, and headache. Barriers included low community pharmacist participation
Watthanathiraphapwong, 2024 (Thailand) [30]	Retrospective	Pre-post study	N/A	6 years	Paediatric Outpatient Setting	Clinical pharmacist	Collaboration within multidisciplinary team	No	Children	Reduction in the number of prescribing errors (443 to 145) and office visits (98 to 21) was observed post-intervention. Prescribing without adequate monitoring also reduced (115 to 14) as well as inappropriate dosage (66 to 14)

### Clinical and medication management outcomes

Across studies, pharmacists were involved in medication initiation, titration, monitoring, and follow-up, operating as independent prescribers [13, 14, 28, 29] in some settings and under collaborative or delegated prescribing models in others [12, 21–27, 30].

Improvements in adherence to physical health monitoring were reported in several studies. In a pre–post college health centre study, pharmacist-led care achieved higher rates of blood pressure (77% vs 11%) and heart rate monitoring (75% vs 6%) than psychiatrist-led care [12]. In a cluster randomised controlled trial, children managed by pharmacists were more likely to meet height and weight monitoring standards compared with paediatrician-led care (88.6% vs 66.1%), with comparable parental ratings of care quality [21]. Furthermore, Shah et al. reported that pharmacist contributions, including monitoring of physical parameters, were positively received by families and multidisciplinary team members [13], while Watthanathiraphapwong et al., reported reduced stimulant prescribing errors and enhanced parental satisfaction in ADHD care management [30].

Pharmacists also supported successful medication titration and stabilisation. In a UK CAMHS service, 85.7% of patients responded to first-line stimulant treatment following pharmacist-led titration [14]. In an adult ADHD service, 67% of patients were stabilised under pharmacist-led care, with an average of 3.7 follow-up contacts per stabilised patient [23].

### Service delivery, efficiency, and access to care

Pharmacist integration was associated with improvements in service efficiency and access. In a UK CAMHS service, waiting times for medication initiation were reduced from seven to three months [14]. In a US college health centre, pharmacist involvement led to a more than tenfold increase in ADHD-related appointments, expanding service capacity and access [12]. Other studies reported reductions in psychiatrist workload and improved clinic workflow following transfer of routine medication management and follow-up to pharmacists [22, 29].

### Economic and resource outcomes

A few studies reported cost savings associated with pharmacist-led ADHD services. In a long-term CAMHS evaluation, pharmacist involvement reduced annual drug expenditure by approximately £133,000, yielding a net saving of £97,000 per year [27]. In a large adult ADHD service, pharmacist-led care was associated with cumulative cost savings exceeding $760,000 over five years, largely attributed to workforce substitution [23].

### Professional contribution and system-level functions

Pharmacists contributed to medicine-related education, clinical governance, and medication safety. They provided advice and education to patients, families, and multidisciplinary teams, and in some settings supported school or college personnel [13, 25]. Several studies reported pharmacist involvement in developing local prescribing guidelines, standardised monitoring protocols, and medicines optimisation processes [14, 22, 27]. Medication safety activities included identification of adverse drug reactions and assessment of potential drug–drug interactions [23, 28, 29].

### Barriers to implementation and sustainability

**Fig. 2:**
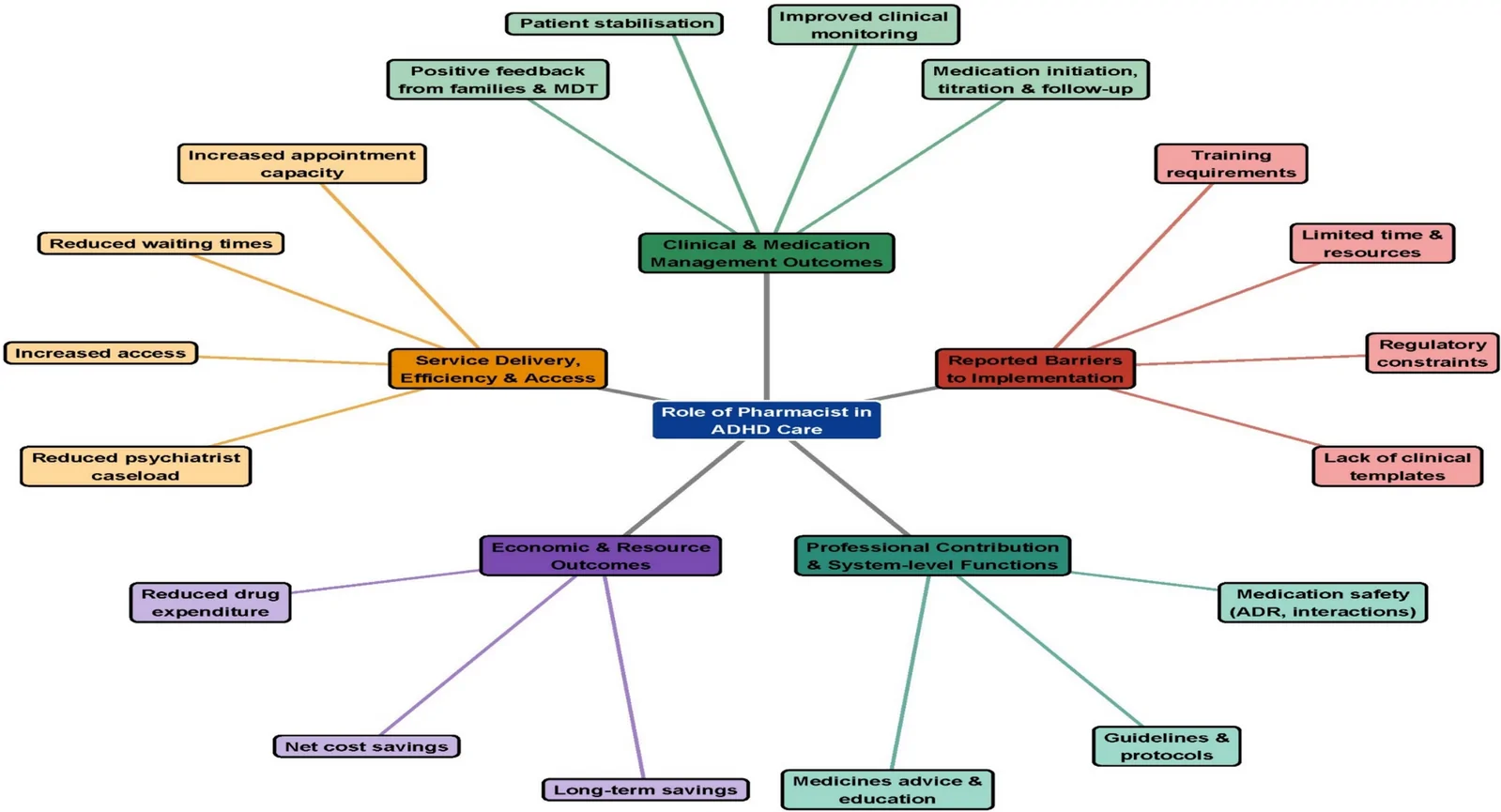
Role of pharmacist in ADHD care

Reported barriers to the implementation of pharmacist roles in ADHD services included lack of structured clinical templates to support early implementation [12], regulatory and scope-of-practice constraints, particularly in US settings [23], and limited reimbursement mechanisms [24]. Additional service-level challenges included constrained pharmacist capacity, low uptake in community pharmacy settings [28], and the time required for specialised training during early service development [29]. A summary of pharmacist roles across included studies is presented in Fig. 2.

## Discussion

### Summary of key findings

This scoping review mapped the existing empirical evidence on pharmacist involvement in ADHD services and identified a small but growing body of literature published predominantly in the last decade. The evidence base is concentrated in the United States and the United Kingdom and is characterised largely by service evaluations, pre-post studies, and post-implementation reports, with relatively few comparative or experimental designs. Across diverse settings and populations, pharmacists contributed to a wide range of ADHD-related activities, spanning clinical and medication management, service delivery and access to care, medicines-related education and governance, and broader system-level functions. Overall, the findings suggest that pharmacist involvement in ADHD care is feasible across multiple care settings, but the nature, scope, and reported impacts of these roles vary considerably.

### Nature and scope of pharmacist roles in ADHD services

Pharmacist involvement in ADHD services predominantly occurred through prescribing and advanced clinical roles embedded within multidisciplinary teams, reflecting the extent to which pharmacists are clinically integrated within local service structures. The balance between prescribing and non-prescribing activities appears less indicative of role capability than of how pharmacists are positioned within care pathways, with variation largely shaped by regulatory permissions and scope-of-practice arrangements across health systems, particularly between the United States (US) and the United Kingdom (UK).

In the US, pharmacist prescribing authority is governed at the state level and implemented through a range of models, including patient-specific and population-specific Collaborative Practice Agreements, statewide protocols, and class-specific prescribing [31]. This regulatory complexity contributes to substantial heterogeneity in pharmacist autonomy and responsibility within ADHD services, shaped by state legislation, institutional policies, and local service agreements.

In contrast, the UK operates under a nationally regulated framework for pharmacist independent prescribing, established in 2006, which permits pharmacists to prescribe any medicine within their area of competence [32]. Despite this enabling framework and the increasing number of pharmacist independent prescribers, including reforms whereby all newly registered pharmacists will qualify from 2026 [33], evidence from this review suggests that pharmacist involvement in ADHD services remains limited, with few empirical studies and no standardised models of practice. Where implemented, pharmacists often worked with a high degree of autonomy in medication titration and monitoring, but these roles appeared locally developed rather than systematically embedded within national care pathways. This gap between regulatory permission and service-level implementation highlights the need for stronger empirical evidence and more consistent models to support workforce expansion and reduce regional variation in access to ADHD care [4].

### Interpretation of reported outcomes

The outcomes used to evaluate pharmacist involvement varied according to local service priorities. Studies embedded within service redesign initiatives focused primarily on operational outcomes such as appointment capacity, waiting times, and workflow efficiency, whereas clinically oriented evaluations emphasised monitoring adherence, medication stabilisation, and follow-up frequency. This pattern suggests that outcome selection was often driven by organisational pressures rather than a shared evaluative framework, resulting in stronger evidence for feasibility and service performance than for patient-centred or clinical effectiveness outcomes.

Comparative evidence was limited; however, where evaluated, pharmacist-led care achieved comparable clinical outcomes to other prescribing models, consistent with broader evidence from chronic disease and mental health care [11, 15, 34]. These findings likely reflect pharmacists’ expertise in medicines management and structured monitoring. However, the small number of comparative studies and the predominance of single-site evaluations constrain generalisability.

Economic outcomes were primarily reported in retrospective evaluations and long-term service reviews, with cost savings largely attributed to workforce reconfiguration rather than changes in medication use or care intensity. In these models, pharmacists assumed responsibility for routine medication management, enabling specialist clinicians to focus on diagnostic assessment and complex cases. This suggests that economic benefits are mediated through role substitution and redistribution of workload rather than direct patient-level effects [27]. While indicative of potential value, further cost-effectiveness analyses are needed to robustly establish these findings.

Furthermore, patient-reported outcomes, such as symptom control, quality of life and treatment experience, were less prioritised. Where patient satisfaction or perceived quality of care was assessed, it was typically secondary to service efficiency outcomes and reported descriptively. This contrasts with the broader ADHD literature, which emphasises patient experience, shared decision-making, and continuity of care, particularly for long-term medication management [35]. Although evidence from other contexts suggests that patient experiences of pharmacist prescribing are generally positive [36], there remains a clear need for ADHD-specific evidence. However, it is important to note that these findings should be interpreted in the context of the underlying study designs. The predominance of descriptive and non-comparative studies limits the ability to draw causal inferences regarding effectiveness, and reported benefits should therefore be interpreted cautiously.

### Child and adult service contexts

Differences were observed between paediatric and adult services. Particularly in the UK context, most studies identified in this review were conducted within children’s services, particularly CAMHS. This likely reflects the historical conceptualisation of ADHD as a childhood condition and the earlier development and relative maturity of paediatric ADHD pathways. Although ADHD is now recognised as a lifelong condition for many individuals, adult services have developed more recently and remain unevenly commissioned and variably accessible [37].

Rapid growth in adult ADHD diagnoses over the past decade has placed significant pressure on services that were not designed to accommodate current demand [38]. Adult ADHD care in the UK is characterised by fragmented pathways, limited specialist provision, and prolonged waiting times, often exceeding those seen in children’s services [8]. These structural differences may partly explain why pharmacist roles have been more frequently evaluated in paediatric settings, where pathways are more established and monitoring requirements are clearly defined. At the same time, this highlights a notable evidence gap in adult ADHD care and during transition from child to adult services, a period associated with increased risk of treatment discontinuity [39].

### Barriers and implications for service development

Reported barriers to pharmacist-led ADHD services included regulatory constraints, limited prescribing authority, inadequate funding or reimbursement mechanisms, and time pressures. These challenges are consistent with the broader mental health pharmacy literature [11]. However, ADHD-specific implementation barriers, particularly those relating to competencies required for safe titration and monitoring, remain underexplored. Addressing these challenges will require clearer role definitions, ADHD-specific training supported by supervised practice, and commissioning models that enable sustainable pharmacist integration. Future research should generate robust evidence on clinical, service, and economic outcomes to support the development of effective and scalable pharmacist-led ADHD services.

These findings also highlight the importance of broader implementation considerations when integrating pharmacist roles into ADHD services. Factors such as training requirements, clinical governance structures, prescribing supervision, and commissioning arrangements (e.g., funding, service planning) may influence the scalability and sustainability of these models but were inconsistently reported across studies. Future research should adopt an implementation science perspective to more systematically examine these contextual determinants and inform the development of scalable service models.

### Strengths and limitations

This review is timely, addressing pharmacist involvement in ADHD care at a point when services are under increasing pressure, and there are growing calls to strengthen and expand provision. By focusing specifically on pharmacist roles, it provides a targeted synthesis with direct relevance to workforce development and service innovation. Methodologically, the review followed established scoping review frameworks, incorporated comprehensive database and conference searches, and included independent verification of data extraction. However, several limitations warrant consideration. The review was restricted to English-language publications, potentially excluding relevant international evidence. Included studies were heterogeneous in design, setting, and outcomes, and were predominantly service evaluations, pre–post studies, or uncontrolled designs, limiting the strength of conclusions that can be drawn regarding effectiveness and causal inference. Similarly, economic findings should be interpreted cautiously, as reported cost savings were largely derived from retrospective service evaluations without formal economic evaluation methods, and therefore do not represent robust cost-effectiveness analyses. Consistent with scoping review methodology, no formal quality appraisal was undertaken. Finally, unpublished service evaluations and local reports may not have been captured, potentially underestimating the extent of pharmacist involvement in ADHD care.

## Conclusion

This scoping review identified a small but growing body of empirical evidence on pharmacist involvement in ADHD services, primarily from the US and the UK. Pharmacists were engaged in a broad range of activities, including medication initiation, titration, monitoring, and patient education, with potential benefits for service efficiency and adherence to monitoring guidelines. However, the evidence base remains limited by heterogeneity in study design, outcomes, roles, and settings, with few comparative studies and limited reporting of patient-reported outcomes or long-term clinical impact.

The findings highlight an emerging role for pharmacists in ADHD care but also indicate that more detailed investigation is needed to clarify the most effective models of integration, the competencies required for ADHD-specific practice, and the contextual factors influencing implementation. Future research should prioritise robust evaluation of clinical, service, and economic outcomes to inform evidence-based service development and workforce planning.

## Supplementary Information

Below is the link to the electronic supplementary material.

Supplementary file1 (PDF 148 KB)

## Data Availability

All data supporting the findings of this study are available within the paper and its supplementary information.
